# Process evaluation of text-based support for fathers during the transition to fatherhood (SMS4dads): mechanisms of impact

**DOI:** 10.1186/s40359-019-0338-4

**Published:** 2019-09-13

**Authors:** Richard Fletcher, Tess Knight, Jacqui A. Macdonald, Jennifer StGeorge

**Affiliations:** 1 Family Action Centre, School of Health Sciences, Faculty of Health and Medicine, University of Newcastle, NSW University Drive, Callaghan, NSW Australia; 2 Centre for Social and Early Emotional Development, School of Psychology, Faculty of Health, Deakin University, Geelong, Australia; 3 Department of Paediatrics, University of Melbourne, Parkville, Australia; 4 Murdoch Children’s Research Institute, Melbourne, Australia

**Keywords:** Process evaluation, Fathers, Mechanisms, Text-based, Qualitative

## Abstract

**Background:**

There is growing evidence for the value of technology-based programs to support fathers to make positive transitions across the perinatal period. However, past research has focused on program outcomes with little attention to the mechanisms of impact. Knowledge of why a program works increases potential for replication across contexts.

**Methods:**

Participants were 40 Australian fathers enrolled in the SMS4dads text-based perinatal support program (Mean age 35.11 (5.87). From a starting point between 16 weeks gestation and 12 weeks postpartum, they were sent a maximum of 184 text messages. An inductive approach was used to analyse post-program semi-structured interviews. The aim was to identify mechanisms of impact aligned to previously identified program outcomes, which were that SMS4dads: 1) is helpful/useful; 2) lessens a sense of isolation; 3) promotes the father-infant relationship; and 4) supports the father-partner relationship.

**Results:**

We identified two types of mechanisms: four were structural within the program messages and five were psychological within the participant. The structural mechanisms included: syncing information to needs; normalisation; prompts to interact; and, the provision of a safety net. The psychological mechanisms were: increase in knowledge; feelings of confidence; ability to cope; role orientation; and, the feeling of being connected. These mechanisms interacted with each other to produce the pre-identified program outcomes.

**Conclusions:**

If the current findings are generalisable then, future mobile health program design and evaluation would benefit from explicit consideration to how both program components and individual cognitive and behavioural processes combine to elicit targeted outcomes.

## Background

Fathers are now recognised as an appropriate target for early intervention services aiming to improve mental health and wellbeing outcomes for families [1–3]. This recognition arises from a body of evidence demonstrating that a father’s health-related behaviours and his relationships with the mother and his child, from the point of conception, up to and after the birth, are important determinants of family wellbeing [4, 5]. At the same time, community perceptions of a father’s role have evolved to include an expectation that male parents will take some responsibility for infant care and nurturing [6–9].

However, while calls regularly appear for family-related services to include fathers alongside mothers, engaging fathers with professional sources of information and support remains a challenge [10, 11]. This is problematic because the transition to parenthood is a period of increased psychological risk [12, 13]. Transitions are a staged physical, psychological, and spiritual process that involve a reorganisation of internal and external life and therefore often characterised by asynchrony and disequilibrium [14–16]. As such, they can perpetuate existing symptoms of problematic behaviours, trigger relapses of past symptoms or behaviours, or precipitate first onset of symptoms or behaviours [17–19]. Transition and its vulnerability are different for fathers compared to mothers because men respond differently to stress, to socialisation, and to approaches to relationship building with the child [20]. The transition period has been described by fathers as a no-man’s land [21], as they are neither ‘not-father’ nor oriented to the new status and practices of fatherhood. Fathers can feel as though they are ‘outsiders’, they may experience conflict with partners, and face competing demands across family, work, and relationships [22]. For some men, internal and external stressors such as these may lead to depression [23].

Studies of fathers across regions using a variety of measures and cut points have established the significance of paternal perinatal depression. A meta-analysis of 43 studies with 28,004 participants found prenatal and postpartum depression was evident in 10% of fathers [24]. The evidence of negative impact of paternal depression on infant and child development clearly warrants attention to fathers as part of a public health approach to improve perinatal mental health [25]. While there are legislative, regulatory and policy motivators for determining the best methods for screening and treating maternal perinatal depression and anxiety [26–28], attention to paternal mental health is relatively new [29].

Fathers’ lack of access to clinics and programs, often due to work commitments, a gendered approach by health service staff to supporting parents, and fathers’ lack of knowledge and reluctance to ask for help have been identified as barriers to engagement with services over the perinatal period [30, 31]. Therefore, novel ways are needed to engage men as they transition to fatherhood. For fathers, online access to information and confidential support via mobile phones at more convenient times addresses some of the barriers to assistance [32, 33]. There is accumulating evidence of benefits of IT-based mHealth programs for parents across low, middle, and high income countries [34–36]. However, there is mixed evidence for effect on parenting practices such as breastfeeding and attendance at antenatal care, prompting researchers to call for more detailed analysis of such programs [34–36]. In particular, it is important to understand the mechanisms operating within programs to keep parents engaged and to produce the targeted outcomes [37]. Qualitative methods can help to elucidate the context and pathways by which a program produces its outcomes [38]. In this study therefore, we aim to qualitatively assess the effectiveness of a mHealth program, SMS4dads, to understand what worked and why.

### SMS4DADS

SMS4dads is a text-based intervention for antenatal and postpartum fathers. The program consists of a set of 184 brief (160 characters or less) text messages delivered at varying days and times addressing a father’s relationship with his baby (*n* = 72), his relationship with and support of the baby’s mother (*n* = 61), and his own self-care (*n* = 49). Messages were developed through a series of consultations with parents and experts in perinatal mental health and parenting (see [39, 40]). The messages were tailored to a father’s perspective, for example, *Talk to your partner about staying home in the early months. Are there ways you can get more leave?* Many of the messages used the ‘voice’ of the baby, for example, *Babies come with personality Dad. Getting to know my personality can make being my Dad much more rewarding for you*. The message content was tailored to gestation and infant age; messages began at 16 weeks gestation and the last was sent at 24 weeks postnatally. Fifty-two of the texts included links to non-profit online parenting resources. Instructions for opting out were included in 26% of all texts. Every 3 weeks an interactive Mood Tracker text asked participants to indicate their current mood by selecting one of five one-click options ‘awesome’, ‘cool’, ‘OK’, ‘shaky’ or ‘bad’. Indications of high distress triggered an escalation process from a national help-line for perinatal mental health support.

A feasibility study of SMS4dads [41] recruited 520 fathers from across Australia through perinatal health services and social media sites. Fathers could enrol at any time from 16 weeks in the pregnancy through to 12 weeks post birth and received texts until their infant reached 24 weeks of age or they opted out. At the conclusion of the messages, 101 fathers completed an online survey indicating high approval of the program: 92.9% found the messages helpful; 83.3% said they felt less isolated as a result of the program; 80.9% found the messages helped their relationship with their partner, and 65.4% with their infant [41].

## Method

Interviews with fathers were analysed to identify possible program drivers of behavioural or cognitive change, specifically those that appear to promote men’s healthy transition to fatherhood.

### Participants and procedure

Participants were 40 fathers who, on completing SMS4dads program, participated in a telephone interview. As part of their final SMS4dads message participants could consent to being contacted to request an interview as part of the project evaluation. Over a 2 month period all eligible participants who consented to being contacted were sent a text message requesting an interview. Of those invited, 58% (43/74) nominated an appointment time, 93% (40/43) of whom went on to complete an interview. Fathers were interviewed by two male research assistants: one whose doctorate had included extensive interviewing with fathers, the second interviewer was a final year psychology student who was trained in interview technique by the last author. The interview questions, following a semi-structured interview protocol were aimed at understanding if and how the program had assisted the men, specifically with respect to, becoming a father, their relationship with their infant, and their relationship with their partner. Interviewed fathers were mostly similar to those who completed the program but were not interviewed, however were less likely to be stressed than those not interviewed (*p* < .05; see Table 1). Fathers’ views were also sought on the timing of messages, types of messages that were better recalled, and any benefits or negative aspects of the program. The recorded interviews, which lasted from 10 to 34 min (average 20.4), were transcribed and entered into NVivo software [42] for management of data records and subsequent analysis. A copy of the interview was offered to all fathers interviewed, 19 requested a copy, and no fathers subsequently requested revisions to their transcripts.

**Table 1:** Baseline sample characteristics by interview condition

	Interviewed (*n* = 40)	Not Interviewed (*n* = 494)	
	Mean (SD)		*t*
Age	35.11 (5.9)	33.57 (5.2)	1.61 (44), *p* = 0.115
	*n* (%)		*x* ^2^
Cultural background			0.02, *p* = 0.883
Non AorTSI	40 (100)	480 (97.2)	
AorTSI	–	14 (2.8)	
Number of children			0.59, *p* = 0.443
First Child	33 (82.5)	428 (86.6)	
One or more	7 (17.5)	65 (13.3)	
Prosperity			2.77, *p* = 0.428
Very poor	–	1 (0.2)	
Poor	1 (2.5)	4 (0.8)	
Just getting by	4 (10.0)	82 (16.6)	
Comfortable	28 (70.0)	294 (59.5)	
Very Comfortable	7 (17.5)	94 (19.0)	
Prosperous	–	16 (3.2)	
Audit-C			0.27, *p* = 0.603
No risk	17 (42.5)	231 (46.8)	
At risk	23 (57.5)	263 (53.2)	
DASS			
Depression			0.00, *p* = 0.995
No risk	32 (80.0)	395 (80.0)	
At risk	8 (20.0)	99 (20.0)	
Anxiety			1.72, *p* = 0.189
No risk	31 (83.8)	401 (90.5)	
At risk	6 (16.2)	42 (9.5)	
Stress			3.86, *p* = 0.049*
No risk	36 (90.0)	378 (76.5)	
At risk	4 (10.0)	116 (23.5)	

Audit-C: Bush, K., Kivlahan, D.R., McDonell, M.B, Fihn, S.D., & Bradley, K.A. (1998). The Audit alcohol consumption questions (AUDIT-C): An effective brief screening test for problem drinking. *Archives of Internal Medicine, 158,* 1789–1795; DASS: Lovibond, P. F. (1998). Long-term stability of depression, anxiety, and stress syndromes. *Journal of Abnormal Psychology, 107,* 520–526

*significant at *p*<.05

### Analysis

Epistemologically, we expect that there is a simple relationship between what the men say about SMS4dads and their experiences and understandings of the program [43]. With this understanding, a deductive-inductive approach to thematic analysis of the men’s interview data was used to categorise the key concepts arising from the evaluation aims and those emergent from the data [44].

The following steps were taken. First, in order to facilitate coder agreement, the texts were unitized [45] such that a selected code would be applied to a whole paragraph of text, since the structure of the interviews resulted in relatively short paragraphs. A coding framework was derived from the evaluation aims and from initial close readings of four transcripts by RF and JSTG. The framework consisted of six upper level categories with subcategories that captured a medium-grained but complete representation of participants’ responses to and interactions with SMS4dads**.** This included their perceptions of the benefit of the program, their comments about their relationship with their partner, comments about their baby, and about their own help-seeking. Commentary about their reactions to specific design features was also coded, such as message scheduling, baby voice, normalisation, and credibility. Overlap and redundancy across the coding categories was avoided; however, the categories within the coding hierarchies were not mutually exclusive, and text could be coded at more than one category, including from the same hierarchy. Category titles, descriptions and exemplar quotes were compiled into a code-book to be used by the researchers to maintain accuracy and consistency.

The four authors then coded (on paper) approximately 10 interviews each, with pairs of coders double coding 20% of the interviews to maintain coder agreement. A team meeting was held half-way through this process to discuss differences and adjust category titles. One new category emerged at this point. At the completion of the paper coding, a research assistant entered all transcripts into NVivo 12, and applied the coding as marked on paper by the authors.

The textual content of the categories was then exported and distributed to the authors for summarisation and synthesis, following Bazeley [46]. The characteristics, frequency (number of paragraphs and data sources coded), and content of each category were described, and then categories were compared in order to ensure distinctness and clarity. In order to explain how the outcomes of SMS4dads occurred in relation to the explicit structural features (that is, explain the mechanisms of impact), and sensitised by concepts within transition theory, the psychological processes observed in the data were classified to create a set of new categories which represented knowledge construction, confidence, coping, feeling connected, and role orientation. Data coded at both a structural feature and a psychological process provided evidence for the findings.

## Results

### Positive outcomes of SMS4dads

Four positive outcomes of SMS4dads were identified in the feasibility study discussed above [41]. These were that the messages: were perceived to be helpful; reduced a sense of isolation; promoted relationship with infants; and, helped relationship with partners [41]. In the current study, we distinguished two categories of mechanisms that appeared to act in combination to generate the positive outcomes found in the feasibility study. The first category comprised *structural features* of the SMS4dads messages. These were explicit design features embedded in the content and timing of the messages and included: 1) evidence-based information synchronised to specific gestational and postpartum needs; 2) normalisation of paternal experiences; 3) prompts to interact and reflect; and, 4) the provision of a safety net (through the Mood Tracker). The second category of mechanisms we distinguished were five *psychological processes* within the participant. These included: 1) knowledge construction; 2) feelings of confidence; 3) ability to cope; 4) role orientation; and 5) a feeling of being connected. In the following sections, we provide evidence of how the *structural features* interacted with one or more of the *psychological processes*, to become mechanisms that produced positive outcomes (Table 2).

**Table 2:** Interaction of structural features and psychological processes mechanisms of impact in SMS4dads

Structural features	Psychological processes				
	1. increase in knowledge	2. feelings of confidence	3. ability to cope	4. role orientation	5. feeling connected
1. synced info	So having that information ready is really a good strategy.	Can interact in ways that are appropriate	So the timing worked really well... it helps when you get a bit stressed	Because the article and stuff and the link went through how that was beneficial, you feel like you’re contributing.	Again, it was just a text message but you’re checking in on me and you’re saying things that actually are happening to me as I’m getting the text messages.
2. normalising	Once or twice one would come in just as she was having a bit of a worse off time and it was just helpful to remember that it’s fairly natural thing for them to go through.	Just having the little reminders that it’s not actually anything we were doing specifically, but it’s just how this whole process works. It definitely helps restore your confidence when you’ve had a hit.	So it sort of like calmed me a little bit, from the perspective of not freaking out or whatever or not – you know, not being too concerned about something that really wasn’t a concern, it was just normal.	Just of planting those ideas that OK, that could be why the child’s crying, it’s not to do with me being a bad parent or anything like that.	I suppose it sort of reinforces in me that she is doing a good job and what she’s doing is perfectly normal and she is going to experience these rough times as well.
3. prompts to interact	Some of those message that were a bit instructive about different interactions or things to try. I definitely tried things after seeing messages.	I think they definitely gave me some other ideas…made me feel more confident.	It’s a prompt for me to raise something that maybe I would have left until things had gotten sort of to the point where it would have been harder.	That’s what triggered something in me to say, “you know what, I really need to be a little bit more active in that area”.	Every time I got it she was like, “What was the message today?” and then we’d have a joke about the video camera being in the corner of the room.
4.safety net	…click on to the links and go through the articles. But I often find it’s better that way because …[people say] “if you’re not doing it the way I did you’re doing it wrong.”	And you get these coming through and you go, well even if it’s not a friend, at least it’s something that’s reminding me that I’m doing a good job.	It felt like somebody just walked into my office, put their hand on my shoulder and said, “Are you okay?” And that’s golden.	“Look after myself first and then I’m able to help someone else”.	A “sort of mate tapping you on the shoulder”,

Data derived from matrix query in NVivo; analytic commentary in manuscript explicates cells where there was substantial data

#### Evidence-based information synced to the paternal perinatal experience

Having access to evidence-based information specifically tailored and synchronised to the stages of paternal experience within the perinatal period allowed men to build up their knowledge, which related to them feeling more confident with the baby, and more capable of coping. These messages also appeared to reinforce the significance of the father role. In this way, the structural feature of ‘evidence-based information synced to the paternal perinatal experience’ interacted with the psychological processes of knowledge construction, confidence, coping and role orientation to generate positive outcomes for fathers.

##### Psychological process: knowledge construction

The information about maternal health and baby development was perceived as helpful by many men. The credibility, consistency and research-based information in the messages was “something I could trust in”, contributing to fathers’ perceptions of the usefulness and “practicality” of the information. “Short, simple, and informative” messages reminded men of “what she’s going through, and the stresses that she may be under”, and could act as a “sort of reality check” on their partner. The perceived credibility of the information also appeared to boost fathers’ capacity to speak with their partner or service providers about the mother’s health or the child’s development: it “helped with a bit of the language of talking” and “highlighted some of the possible questions”. Fathers often shared information with their partner, who many times would corroborate the information, thus enhancing fathers’ trust in their new knowledge.

Because the messages were synchronised to the baby’s development over the perinatal period, men gained knowledge about their developmental stages, and how to interact with them. Fathers pointed to information about infant sight and hearing, sleep and breastfeeding as useful in giving them an understanding of what to expect. Relevant information would “pop up at the right time”, when fathers might have had “questions that need answers” or needed a refresher, “Hey, now’s the time to start thinking about this”...

Just recognising the different cries or that they do have different cries. That actually triggered a whole night of research. And it was really good. Because of that, I actually learned a whole heap of stuff that I didn’t even know about. (T38GG4)

The messages also helped fathers understand the infant’s mind: texts delivered in the voice of the baby helped to give insight into the baby’s mind and needs, because “you don’t know what they are thinking”. Fathers learned that “it’s her only way of communicating” and that the baby was not “trying to manipulate or annoy you”; as one father stated, “that was a really good sort of grounding message”.

##### Psychological process: feeling confident and able to cope

The knowledge gained through the synchronised father-focused information also operated to encourage men to confidently interact with the baby. The “simple, informative” messages were perceived to give guidance and clarity, helping fathers be more sure of how and when they could interact “in ways that are appropriate”. Many fathers reported enjoying the messages that described how their interactions benefited their child and they described multiple ways in which they applied their new learnings.

Things like a few suggestions about ‘go take your baby for a walk’. Then halfway through, I was getting bored of his walks. Then I got a message, for example, that as they grow, they look at things differently and they process information differently. So I continued do those walks with him because he's seeing things in a different way. (MH09PY)

The timed synchronised information also helped fathers to cope by putting into perspective issues such as his partner’s health or child behaviour, “changing the frame on how you, you know, the lens at which you see it”. Some of the messages that ‘stuck in’ fathers’ minds were about stepping back, “instead of pushing through”, and having the right information helped fathers to cope.

So the timing worked really well. And I've got to say having, as I said before, like having that one pop up to just remind me that bub’s not like grizzling and crying out of spite for me, it’s just that she needs something…it helps when you get a bit stressed. (TM9C7D)

I’d say it's something to help you stay on track. To get through a really tough phase in your life and, you know, interact, be able to get reminders of what the baby's going through, what your partner's going through and how best to cope with the situation. (WI7LFQ)*Psychological process: Orienting fathers to the paternal role*

The father-specific design features appeared to contribute to fathers’ emerging understanding of the paternal role. Men described that the information alerted them to their specific influence and responsibilities, for one father, because “I massively underestimated my role in the key period of his development”. The information helped keep fathering “front and centre of mind”, others indicating that it “reset” or “re-orientates”, back to “what’s probably more important”. Some fathers said that the texts gave them purpose, helped them feel ‘included’, ‘important’ and ‘valued’ and gave them ‘ownership’ of the role, thus reducing their isolation. The confidence gained enabled the men to see depth in their role as father and partner, and their capacity for genuine contribution to the family, rather than remaining on the periphery, responding to requests.

That’s something that I can be doing, especially as Dad. And when Mum needs some time…that’s something that I can do. And because the article and stuff and the link went through how that was beneficial, you feel like you’re contributing. You’re not just the drinks guy go getting her a cup of tea or making dinner or cleaning up. You’re actively doing something for the baby. (YEC00D)

As a father you're trying to balance the baby as well as work and as well as any other factors. So, it, I guess in a good way, it re-orientates you to what’s probably more important. (92TLSH)

Overall, the structural feature of ‘credible information synced to the paternal perinatal experience’ interacted with four cognitive-psychological processes to create mechanisms that generated positive outcomes for fathers’ relationships with their partners and their new baby.

Like I said before knowledge is power. It’s about empowering dads to take an interest and to arm themselves with that kind of information right, because that’s what is going to help you. If you go in blind and you don’t know anything, you’re much more I feel, you’re much more likely to potentially struggle and to feel that powerless and to not be able to you know help or assist. Whereas every time you read something and you know like I would read it in an SMS. I would read it in an article, I would see it on a dad advice video, I would talk to [my partner] about it. And it just reinforces that information. (YEC00D)

#### Normalising

A proportion of the SMS messages were constructed to inform fathers about typical occurrences throughout the perinatal period, for example, difficulties in breastfeeding, crying, sleeping or mood. With the intention of normalising men’s experiences, these types of messages conveyed to an extent the likelihood and prevalence of the issues. As a structural feature therefore, this normalisation mechanism appeared to instil in men a confidence in their own ability to understand and respond to their baby’s needs. Relatedly, the normalisation appeared to assist fathers in coping with difficulties. Thus, this structural feature interacted with the psychological processes of confidence and coping to generate positive outcomes for fathers.

##### Psychological process: feeling confident and ability to cope

Fathers stated that the messages offered reassurance and “peace of mind” by giving reasons for the baby’s behaviours, and suggesting adaptive ways in which to respond, preventing fathers “freaking out”. Learning that many phenomena were typical, was helpful, “particularly in the early days with that purple crying and stuff. I thought the texts were good, just another angle to reinforce to say look this is normal, here’s some things to do.” Fathers felt that having the information as “reminders” meant that they could “take it in your stride”, knowing what was expected in preparing for the birth, or once out of hospital, for example. The normalisation messages gave a positive frame to men’s experiences, helping them realise that “the things that I’m going through are not, I suppose, unique or isolated just to me.”

The normalising messages also confirmed fathers’ confidence in their own and their partners’ caregiving strategies. The content of the messages affirmed their ability to be a good father, “I’m not such a Gumby at this stuff”, acting as a type of feedback, giving reassurance and confirmation that parents were on the “right track”, and that “it’s not to do with me being a bad parent or anything like that. You know, this is what a normal kid does.”

The normalising messages also assisted fathers to cope, when feeling “lost” or “wary” for example, or “after a hard night”, because “if you know that there’s other people that have the same sort of issue, it’s easier for you to deal with it.” Such messages could also prompt discussions between partners that then enhanced their coping ability; the messages were reminders for them both that this is “how this whole process works.” One father talked about how the normalisation helped his wife, “sort of changed the conversation from, ‘I’m failing as a mum’ to, ‘it’s out of my hands, it’s just everyone goes through it’.” In a sense, these normalising messages helped to reframe fathers’ worries from problems to normal concerns for first-time fathers.

#### Prompts to interact

A proportion of the messages focused on opportunities for interaction and connection. These messages were designed as reminders, prompts or instructions to act or reflect, by ending with a question, for example, “can you find a way to show her this is true?” This structural feature interacted with psychological processes of connection and role orientation to generate positive outcomes for fathers.

##### Psychological process: feeling connected

Embedded in the messages, the prompts to act and reflect interacted with fathers’ feelings of connection to their partner and baby. The prompts opened lines of communication between partners, sparking positive conversations about sleep, crying, or shared household labour. The conversations helped some feel knowledgeable, “she’s not the sole source of information”, and facilitative, “I can show her some of the messages and show her that what she’s feeling and what she’s thinking isn’t abnormal.” Through the act of sharing this normalisation message, mother and father could be “on the same page”, both seeing the importance of talking and working together. By acting on these messages, fathers could show their partners that they were supportive, “involved” and “actually be more present and helpful”.

The messages also prompted fathers to take up “difficult conversations” with their partner, to discuss struggles and ameliorating or avoiding conflict, “[the texts] helped in smoothing it out and not causing a lot of conflicts or avoiding a lot of conflicts that probably could have been there.” As well as avoiding tension, the prompts gave parents something to laugh at together:

She loved them too; every time I got it she was like, “What was the message today?” and then we’d have a joke about the video camera being in the corner of the room. (2HPDPL)

##### Psychological process: orienting fathers to the paternal role

The texts as prompts to interact with the baby, or to be proactive in supporting the mother, also triggered fathers’ thoughts about their paternal role. The prompts pointed to the new practices of fathering, helping to draw the “bigger picture” and orienting them to “what’s important”, “what am I doing squaring a network?” The messages showed men how they might “take a more active role”, and motivated them to consider their responsibilities:

And I think messages that really resonated with me, and made me think more about what I've got myself in to is when you've said, one of the messages says, like ‘how will you make time for me, Dad?’, so it's putting me in that role before I'm actually in that role. (WUGX5S)

One father explained how the prompt to interact with the baby sharpened his realisation that he has an important role, that he is “actively doing something for the baby, and that was good”. Even when men felt they were “pretty aware”, the messages “keep you on your toes”, prompting action when “you know you haven’t been doing it as much as should have.”

That’s what triggered something in me to say, ‘you know what, I really need to be a little bit more active in that area’. (T38GG4)

#### Safety net/virtual mate

The *safety net* was a structural feature that interacted with psychological processes of coping and connection. Messages about self-care were designed to provide information about men’s mental health, and through the MoodTracker, to prompt reflection on their own mood and wellbeing. These types of messages appeared to put a floor under the vulnerability that many men were experiencing. The messages were experienced as a “checkup”, “check in” or “pick-me-up”, giving “a bit of hope” and providing resources during a difficult time; presenting the information in an objective yet supportive way.

##### Psychological process: ability to cope

Fathers commented that the texts “really helped, particularly with the mental side of everything”, to “relieve tension from your day” and when “stressed out”. For some men, this was particularly important because “we don’t really get a lot of support as dads, especially in the early phase, and we just always expected to do, to figure it out. And this just gives us a little hint, a little check in”, where fathers learn to “look after myself first and then I’m able to help someone else”.

Some men reported finding it difficult “getting through” but were reassured by the timely and relevant checks. They valued having someone checking in with them and simply asking the question “Are you alright?” When one dad felt “kind of lost” the texts prompted him to “go, ‘oh no, we’re actually doing a pretty good job.’” Another father stated that the texts “make sure that guys know that it’s okay to be feeling overwhelmed”. The texts stimulated reflection: “it causes you to reflect on your own state of mind,” and “think things aren’t that bad or if you have a really good day it actually goes and forces you to acknowledge that”.

The messages were good just as a bit of a support pillar because a lot of the things I had done with her were sort of done during the fog of, not the fog of war but the fog of fatherhood. (T38GG4)

The messages supported fathers when midwives or other professionals were no longer available, and provided links so “if it gets really hard, you know where to go”.

For some fathers, the messages were useful reminders about safe drinking strategies; the texts “made me think” that it’s not a “good thing”; while “only an SMS”, the texts were “a constant reminder that, you know, it’s not acceptable anymore to do that.”

So I’ll give you one example where I was struggling and I needed a bit of time for myself. I was off to the pub, we have a pub that’s about a 20 metre walk from my apartment, not that I drink too much or anything, but I was just like, “I just need a break and I need to go and have a drink,” and I got a text message immediately saying, something along the lines of, “How many units of alcohol are you drinking? Alcohol isn’t the answer, blah, blah, blah”. (2HPDPL)

##### Psychological process: feeling connected

For some fathers, SMS4dads was experienced as “someone”, a “sort of mate tapping you on the shoulder”; thus, an anthropomorphising of the intervention. This interpretation of connection appeared to mitigate the risk of isolation and vulnerability. Although it was understood that the messages were “external support” that came from a “bot”, some men referred to the messages as from “someone”, as if a “faceless person is looking out for you.” “It was just a text message but you’re checking in on me”, there was a sense that there was someone “else” supporting the father:

Sometimes you’re looking for support, and you're looking for someone just to get in touch with you and just be like, “Hey, how are ya?” And you get these coming through and you go, well even if it's not a friend, at least it's something that's reminding me that I'm doing a good job. (3L3F7L)

The attribution of a persona to the messages also appeared to create a sense of anticipation that kept men balanced and in the loop, “Put it this way: if I wasn’t receiving the message, I’d miss them”, because “you’re the only one who’s texted me saying how are things going.”

## Discussion

The aim of this study was to qualitatively assess the effectiveness of the SMS4dads intervention to understand the ways in which it did or did not work. Exploring the ways in which interventions bring about change has been described as crucial to understanding the operation of a particular intervention and how these effects may be replicated [38]. Reproducing or scaling up of interventions is dependent not only on systematic documentation of implementation, but also on a deep understanding of the pathways or mechanisms by which the effects are produced [47].

The analysis of fathers’ interviews distinguished two categories of mechanisms, structural and psychological, that appeared to act in combination to explain the possible effectiveness of SMS4dads in supporting fathers-to-be in their transition to fatherhood. The interviews resulted in sufficiently rich data to allow a nuanced analysis of the complexities involved in a multiple message program stretching across a key period of change for fathers and their families. The deductive-inductive approach to analysis was highly effective in developing coding and schemas to detect candidates for the mechanisms involved. Structural design features of the program matched to outcomes were: 1) evidence-based information synchronised to specific gestational and postpartum needs; 2) normalisation of paternal experiences; 3) prompts to interact and reflect; and, 4) the provision of a safety net. These features interacted with psychological processes within the participant: 1) knowledge construction; 2) feelings of confidence; 3) ability to cope; 4) role orientation; and 5) a feeling of being connected. We consider these mechanisms within three theories: transition theory, social cognitive theory, and therapeutic alliance. Each provide a theoretical framework for understanding the key features of web and text-based programs, and specifically those that aim to improve family outcomes by supporting men as they become fathers.

### Reaching men across the transition to fatherhood

At the instant of conception, men enter a new state of fatherhood, and soon engage in a set of new fathering practices [48]. In this transition, an individual father may be vulnerable because he does not have the knowledge, coping skills, emotional familiarity with the order of things, or the culture and practices of his new status. A smooth journey through this significant transition is facilitated by knowledge and preparation, and positive personal meanings related to the transition [16]. Some individuals have enough resources and capabilities to pass through the transition with little disequilibrium, while others have fewer resources and are therefore vulnerable to mental and physical exhaustion.

However, a feature of mobile technology health interventions is their ability to provide information or support ‘just in time’, that is, at the time when the person receiving the intervention is most receptive to the information or suggestions being conveyed [49]. Men whose partners are pregnant will face a set of challenges over the following years that are by and large predictable. Foetal and infant development are well understood; sensory and motor development, feeding, crying and sleeping capabilities and behaviours that evolve through the perinatal period and are known to lead to parental stress can be mapped chronologically with some precision. The message content of SMS4dads is therefore able to be keyed to frequently experienced paternal challenges based on knowledge of the expected date of birth. The flexibility of the delivery and reception of the messages facilitated active experiential learning, allowing fathers to solve problems as they arose and engendering confidence and connection. Of course, variation in gestation will render some messages inapplicable, as they will refer to infant behaviours or issues that have already occurred or may be delayed. Men’s reaction to receiving messages that did not fit their situation reinforced the importance for fathers of ‘just in time’ information.

### Supporting fathers’ identity and self-efficacy

Other design features of SMS4dads texts enhanced the father’s identity by emphasising the salience of fathering through offering positive appraisal of his role and through increasing his fathering self-efficacy [3, 50]. The normalisation of the fathers’ experiences as a design feature was enhanced by positioning of the program as ‘for dads’. Over the perinatal period fathers will encounter, or become aware of, multiple services aiming to assist new mothers even when promoted as ‘for parents’ [1, 51]. The branding of SMS4dads underlines the legitimacy of fathers’ involvement in the direct care of their infant and the salience of the fathering role. The emphasis on the father-infant relationship, featured in the video promotion of the program and in the message content, is in contrast to the usual representation, found both in theory and in practice, of fathers’ influence operating through the mother-infant relationship [52, 53].

External appraisal of his fathering role was also supplied through the prompts to action that were delivered in the ‘voice’ of the baby. These messages appeared to create a virtual conversation between baby and father where the father is urged to engage with the ‘person’, that is, the infant who is addressing him. In this way the father is called on to take responsibility for the wellbeing of his infant and is recognised for his paternal contribution to the health and wellbeing of his infant. Both the virtual conversation and the suggested actions encourage fathers to seek time alone with their infant, a hallmark of high-level father involvement [54]. Providing texts with prompts to action that embody fathers’ direct care of the infant, and emotional support for the mother, strengthens men’s role salience which, in turn, may increase self-efficacy and involvement [3]. The tailoring of prompts to the timing of postpartum weeks also encouraged fathers to interact in ways that were aligned to the infant’s specific and changing developmental needs. This is important in a context of often ambiguous cues from infants.

### Therapeutic alliance

As flagged in the introduction, paternal mental health has relatively recently been identified as an important factor in family wellbeing and successful infant development. The ability to reflect on his experience, assess his mental health and ask for help in the face of significant distress is a part of fathers’ coping with the transition. When fathers are asked at regular intervals if they are “travelling OK?” in the anonymous format of a text message, SMS4dads normalises reflecting on their mood or mental health. At the same time, the cumulative effect of regular text messages focusing on and validating the difficulties faced by fathers in their transition period can be seen as providing a therapeutic element to the program. Even though the entire program is based on unidirectional message content with no invitation to respond and the sole interactive component, the Mood Tracker, results in professional communication only in cases of self-identified serious distress, fathers anthropomorphise the program and attribute to it an affective and collaborative stance as found in client–therapist relationships [55]. As measures of a therapeutic alliance have been shown to partially explain effects of a group-parenting program on fathers’ parenting competence [56] the potential for fathers’ mental health to be aided via text-based automated programs warrants further investigation.

In the conduct of the study we followed recognised qualitative analysis criteria that facilitated a rich synthesis of deductive and inductive approaches [44, 57]. As reported above, the group of fathers interviewed were similar in their demographic descriptors to the bulk of the fathers enrolled in SMS4dads. We acknowledge the limitations of the data available for matching interviewees with those who did not agree to be interviewed so that there may well be unreported differences, in ethnicity for example, between the fathers interviewed and the larger group of fathers in the program. Although the messages were screened and if necessary reworded to ensure that they required a literacy level below Year 9 [58] the literacy demands of the program may also have excluded some fathers. There were a number of fathers (approximately 20%) who exited before the program conclusion and so were not eligible to be invited for interviews. It is possible that the mechanisms discussed here may not apply to these fathers. As well, the relatively homogenous nature of fathers registering for SMS4dads, in terms of socioeconomic position, limit the generalisability of our findings.

## Conclusion

The focus on the mechanisms of impact of the mHealth SMS4dads intervention responds to the Medical Research Council’s (MRC) concern for the lack of such focus in RCTs [38]. The call for attention to mechanisms necessitates consideration of how elements of an intervention might interact with each other and with contextual factors. In this study, context heightened the importance of providing a program specifically for fathers, whereby the program promoted and validated men’s role as father where they have traditionally felt disempowered. This was also in recognition that fathering occurs in an evolving context marked by rapidly changing developmental needs of infants and by shifting priorities within intimate relationships. The study demonstrated that structural elements of the program that scaffolded the associated psychosocial changes inherent in fathers’ new role. Of importance is an iterative process of testing and redesigning, particularly in the realm of mHealth where technological change is rapid. The study represents a case in identifying supra elements and points to the benefits for future mHealth programs to consider how both structural and individual processes combine to elicit targeted outcomes.

## Data Availability

The dataset used and analysed during the current study are available from the corresponding author on reasonable request.
